# Case Reports Study on Methanol Poisoning in King Abdul Aziz Specialist Hospital, Taif, Saudi Arabia

**DOI:** 10.3390/jcm12134282

**Published:** 2023-06-26

**Authors:** Ghadi I. Alqurashi, Fahad S. Alqurashi, Khalid M. Alhusayni, Alaa H. Falemban, Yosra Z. Alhindi, Safaa M. Alsanosi, Abdullah R. Alzahrani, Saeed S. Al-Ghamdi, Nahla Ayoub

**Affiliations:** 1 Pharmacy Department, King Abdulaziz Hospital, Taif 26521, Saudi Arabia; 2 Saudi Toxicology Society, Umm Al-Qura University (UQU), Makkah 21955, Saudi Arabia; 3 Department of Pharmacology and Toxicology, Faculty of Medicine, Umm Al-Qura University (UQU), Makkah 21955, Saudi Arabia

**Keywords:** methanol poisoning, case reports, metabolic acidosis, visual loss, multi-organ failures, Saudi Arabia

## Abstract

Methanol poisoning is a challenging issue due to its inducing acute multiple organ failures, and especially due to a lack of preparedness, available antidotes, and management protocols. The current study presents six cases of methanol poisoning that attended the emergency department of King Abdul Aziz Specialist Hospital, Taif, Saudi Arabia, between March and November 2022. All of the patients suffered from severe metabolic acidosis and visual impairment following the ingestion of homemade alcoholic beverages and colonia. Three patients were comatose, suffered from post-cardiac pulmonary arrest, and, finally, died, while the other three were non-comatose and discharged from the ICU after improvement. Management was based on clinical symptoms and other laboratory findings due to a shortage of methanol level measurement resources. The antidote, fomepizole, was not given to all of the cases due to its deficiency, and ethanol was given only to one patient due to difficulties in administering it without monitoring its concentration. Methanol poisoning and its outbreak provide insights into the dangers of hazardous homemade alcohol and other pharmaceutical preparations that might be adulterated with methanol, particularly to the shortage of suitable diagnostic testing and antidotes in addition to poor resources for management of intoxicated patients in some regions of Saudi Arabia.

## 1. Introduction

Methanol (colonial spirit) belongs to the alkyl alcohols family, which is the simplest form of this family, and its toxicity occurs through exposure via inhalation, ingestion, or dermal contact with methanol-containing formulations. Methanol is considered most dangerous when ingested. Unfortunately, only 10 mL of pure methanol can be lethal or can cause life-threatening poisoning and permanent vision loss [1]. Methanol is similar to ethanol and is used for many industrial purposes such as airplane fuel, antifreeze, and wiper fluids, in addition to its use as a solvent in cologne, the perfume industry, and homemade formulations [2,3]. Ingestion of methanol may occur accidentally, due to suicide attempts, or adulterated wine [3,4]. Alcohol dehydrogenase (ADH) and aldehyde dehydrogenase (ALDH) are the enzymes responsible for the metabolism of methanol. ADH degrades methanol into formaldehyde, which in turn is converted to the highly toxic formic acid by the action of ALDH [5]. The accumulation of formic acid triggers metabolic acidosis by its self-deposition and inhibition of mitochondrial cytochrome c in addition to multiple organ failures [6]. Furthermore, formic acid accumulation in the eye causes blurred vision and finally visual loss due to the lack of retinal mechanisms of formic acid detoxification [7]. It has been reported that death from methanol poisoning may range between 8 and 36% [8], and patients who still survive after acute methanol poisoning showed permanent vision loss in about 20–40% of cases [2,3,9]. Methanol displays acute toxicity rather than chronic toxicity, which is manifested by gastrointestinal symptoms, central nervous system (CNS) suppression, confusion, coma, and cardiopulmonary arrest, in addition to vision loss and severe metabolic acidosis, the main cause of mortality [10,11]. Fomepizole or ethanol is the standard antidote for methanol poisoning, acting as ADH inhibitors, preventing end-organ damage by inhibiting the formation of formaldehyde and formic acid, in addition to renal replacement therapy [12].

Many social and cultural factors influence the lack of reporting of methanol poisoning cases in Saudi Arabia. Some presentations were delayed because patients feared legal consequences and deferred their visit to a healthcare facility until symptoms worsened and became unbearable, or until they feared permanent vision loss [11,13].

This study clearly points out the limitations of the treatment options in Saudi Arabia (i.e., availability of ethanol/methanol testing in blood in hospitals and antidotes (ethanol/fomepizole infusions)), in addition to showing laboratory test limitations and time constraints. Further, the lack of national treatment guideline protocols and diagnostic criteria of methanol poisoning result in treatment variation. Herein, we report six cases of methanol poisoning in the period between March and November 2022 in King Abdul Aziz Specialist Hospital, Taif, KSA, and discuss the different challenging procedures and issues facing these cases from the public health perspective.

## 2. Material and Method

Six middle-aged patients were admitted to the emergency department, King Abdul Aziz Specialist Hospital, Taif, Saudi Arabia, after alcohol consumption from March 2022 to November 2022. Ethical approval from the Directorate of Health Affairs, Taif, Research and Studies Department, IRB registration number with KACST, KSA: HAP-02-T-067, approval number: 517, date: 12 February 2021 was documented. In this report, the demographic characteristics, laboratory parameters, clinical symptoms, management procedures, and outcome of methanol poisoning was recorded for each case. Written informed consent was obtained from the patients to publish these case reports in accordance with the journal’s patient consent policy. For patients who were unable to give written consent or deceased, it was obtained from the patients’ next of kin.

### 2.1. Cases Presentation

Six methanol poisoning cases were transferred by ambulance to the emergency department of King Abdul Aziz Specialist Hospital, Taif, KSA. Three of them were admitted on 16 November 2022 (two Saudi and one Indian). Another three intoxicated patients were admitted on 4 March 2022 (two Indian, and one Nepalese). The first three patients were comatose, suffered from severe metabolic acidosis, vision loss, and pulmonary cardiac arrest. One of them was hypothermic and another one had acute renal failure and anorexic brain damage. The management of the three comatose intoxicated patients failed and they died. The other three came in together and were non-comatose, drowsy with metabolic acidosis, had toxic optic nerve damage, and one of them was feverish. They were discharged from the hospital after the appropriate management, where they were administered with the antidote fomepizole and hemodialysis in addition to correcting their metabolic acidosis with sodium bicarbonate. Detailed histories were taken from the patients and their companions. All of the cases had failed to buy alcoholic beverages. Instead, they ingested a homemade alcoholic beverage containing 70% ethanol and colonia. They drank several glasses of this alcoholic beverage the day before they were referred to the emergency department. All of the patients were promptly admitted to the intensive care unit (ICU). The demographic and admission profile data for the six cases are presented in Table 1, while their vital signs and clinical laboratory parameters data are presented in Table 2. In addition, the methanol poisoning symptoms and management course data are presented in Table 3 and Table 4, respectively. Plasma osmolality is determined mainly by sodium (Na), its counter ions, and uncharged species such as glucose (GLU) and urea (UN). Knowledge of the plasma concentration of these species allows calculation of the plasma osmolality quite accurately. The difference between the measured osmolality (MO) and calculated osmolality (CO) is known as the osmolar gap (OG). A large positive (>15) osmolar gap can help to identify the presence in plasma of substances such as ethanol, methanol, isopropanol, ethylene glycol, propylene glycol, and acetone. The proper interpretation of the OG also requires knowledge of the anion gap (AG = Na − HCO_3_ − Cl), the blood pH, and qualitative testing of the plasma ketone bodies (KETO). When the OG is combined with the blood pH and AG, poisoning with toxic alcohols can be quickly recognized. The presence of low blood pH, elevated AG, and greatly elevated OG (>15) is a medical emergency that requires prompt treatment [14]. Based on this, the anion gap (AG) was calculated from the following equation: AG = (Na^+^ + K^+^) − (Cl^−^ + HCO_3_^−^) in mmol/L, where a normal range of AG should be 13 ± 8 mmol/L [14]. The osmolal gap (OG) was calculated from the following equation: Serum osmolality–calculated osmolality ((2 × [Na]) + (glucose, in mg/dL)/18 + (blood urea nitrogen, in mg/dL)/2.8) in Mosmol/kg, where normal individuals should have a value between 10 and −10 [15], as illustrated in Table 5. Further, outcome data for the six cases are presented in Table 6.

### 2.2. Final Diagnosis

Methanol poisoning was confirmed in the six cases based on the signs of metabolic acidosis, vision loss, optic nerve damage, and the drink brought by their relatives. The methanol level was measured only for patient 2, while patients 1 and 3 died before sending the samples to the toxicology center in Makkah (methanol was measured by GC/MS Shimadzu, Poison Control Center Makkah, Saudi Arabia). The methanol levels of patients 4, 5, and 6 were not measured and treatment started with the empirical treatment of methanol intoxication.

#### Treatment

Not all of the patients were treated with the appropriate antidotes, such as fomepizole, due to the shortage of its availability in addition to the lack of efficient blood methanol measurement in the hospital’s laboratory. Treatment with ethanol was applied to patient 3, due to the unavailability of fomepizole, and because of the difficulty of administering it without monitoring its concentration, the treatment with ethanol of patients 1 and 2 could not be applied or even continued for patient 3. Most of the patients were treated with supportive care, inotropic support, vitamins (thiamin), and hemodialysis if possible. Hemodialysis was performed on patients 4, 5, and 6 while failing in patients 1, 2, and 3 due to their circulatory collapse.

### 2.3. Follow-Up and Outcome

Three patients died from post-cardiac pulmonary arrest after ICU admission. In addition, patients number one and three rapidly died from multi-organ failure 10 h after ICU admission. Patient number one showed diffused brain edema and anorexic brain damage in the CT scan in addition to acute renal failure and other symptoms of methanol poisoning, while patient number two died two days after ICU admission. The other three patients survived and were discharged five days (patient 4), and 3 days (patients 5 and 6) after ICU admission but with permanent optic nerve damage.

## 3. Discussion

Outbreaks of methanol poisoning have been described in the medical literature in different regions around the world, in particular in Arab countries [3,7,16,17,18,19]. Even though in Saudi Arabia a few outbreaks of methanol poisoning have occurred [20], they remain undocumented. Herein, this article describes six cases of methanol poisoning in Saudi Arabia with the goal of increasing awareness about the dangers of methanol poisoning among healthcare staff.

In this study, we described the clinical symptoms, management procedures, and outcomes of six methanol-intoxicated patients managed in Taif, providing an insight into the limitations that have affected the treatment strategy and reviewing fundamental public health issues in KSA that remain unresolved to date.

All six patients developed severe metabolic acidosis, the most common finding of methanol poisoning, due to formic acid and formaldehyde accumulation [6,7]. In addition, each patient had visual disturbances, the only specific characteristic of methanol poisoning. Metabolic acidosis coupled with visual disturbances from methanol poisoning had been reported in several studies, with a prevalence of approximately 30–60% on hospital admission [21,22,23]. In this study, all cases reported visual loss and toxic optic nerve damage. This is in agreement with a previous case series study in KSA at the King Khaled Eye Specialist Hospital and King Saud University Hospitals in Riyadh, that reported that 100% of cases showed visual loss due to bilateral optic nerve damage, which is a late complication of methanol poisoning, while other symptoms reported in the current study were not assessed [3]. Formic acid accumulation in the eye causes blurred vision and finally vision loss due to the lack of retinal mechanisms of formic acid detoxification [5,7]. Brain damage was also present in our cases, where only patient 1 reported diffused brain edema and anorexic brain damage, as seen in the CT scan. This patient with brain damage had shown more severe methanol poisoning and more severe metabolic acidosis than the other patients, without brain damage. A previous study supported this finding, where brain damage and lesions were present in severe methanol poisoning cases with in patients with the highest metabolic acidosis [24].

All cases were suspected to have methanol poisoning based on their symptoms and history of alcohol and colonia ingestion. After ingestion, methanol may persist in the body for as long as a week. It is water-miscible and distributes in total body water, therefore, higher levels are attained in the aqueous and vitreous humors of the eye, CSF, and gastric secretions than in blood [16,25]. Its volume of distribution ranges between 0.60 and 0.77 L/kg [16,26]. The first indicators of methanol intoxication are difficult to distinguish from normal ethyl alcohol effects within an hour, with mild symptoms such as nausea, vomiting, and abdominal discomfort being similar to ethanol poisoning. Following a latent period of approximately 12–24 h, depending on the methanol dose consumed, metabolic acidosis develops, and visual function deteriorates, ranging from blurred vision and impaired visual fields to full blindness, side by side with symptoms such as headache, dizziness, and vertigo. Worst of all, it takes 12–24 h to distinguish between methanol and ethanol poisoning, and people are frequently heavily intoxicated and unconscious [16,26].

Once methanol poisoning is suspected, based on their symptoms and alcohol ingestion history, their serum methanol must be measured, and this was not available for all six cases. Hence, the osmolality gap and anion gap metabolic acidosis should be evaluated as soon as possible to confirm methanol poisoning. The anion gap (AG) and osmolal gap (OG) have been reported as necessary tools in the evaluation of methanol poisoning and for guiding treatment procedures [14,19]. It has been reported that a high AG and OG are correlated with severe and non-specific metabolic acidosis, with secondary respiratory dyspnea as a result [26]. The AGs and OGs for the six cases were not calculated in the hospital. However, in the present study the authors used the available data to calculate the AG and OG for the purpose of evaluating the poisoning by methanol and the treatment procedures, which did not follow a specific protocol (Table 5). The current study reports that those patients who died had higher AGs and OGs, compared to those who survived (*p*-value = 0.037 for AG and 0.024 for OG).

Metabolic acidosis is the main symptom of methanol poisoning and leads to other symptoms and complications of methanol poisoning. However, rapid correction of metabolic acidosis and elimination of formate is necessary and a cornerstone of methanol poisoning management procedures [12,27].

Different antidotes have been reported to be used in the management of methanol poisoning, trying to lower levels of formate, released from methanol metabolism, including ethanol and fomepizole [28]. Fomepizole, is an ADH inhibitor, with a longer duration of action; it prevents the conversion of methanol to its toxic metabolite formic acid [28]. It is given intravenously at a loading dose of 15 mg/kg within 30 min, then 10 mg/kg every 12 h until the methanol level drops below 30 mg/dL. To overcome p450 enzyme clearance, the dose of fomepizole should be raised after 48 h. Because hemodialysis also removes fomepizole, the dosage should be adjusted during the hemodialysis procedure [28]. Fomepizole is recommended to be given when patients show severe metabolic acidosis with high AG, OG, visual defects, and serum methanol > 20 mg/dL [5,29,30]. In this study, it is noted that only three patients received fomepizole, while the other three did not meet the aforementioned criteria for the use of fomepizole.

Ethanol, another antidote for methanol poisoning, acts by competing with methanol for ADH; it is used in case of the unavailability of fomepizole and it has been reported to be sufficient in preventing formate formation [1,31]. Every 1–2 h during methanol poisoning treatment, serum levels of ethanol must be monitored. The level of ethanol in the blood should be controlled within the range of 100–150 mg/dL to accomplish the therapeutic aims [32]. A loading dose of 600 mg/kg (13 mmol/kg) is initially given to attain this goal. The maintenance dose (66–154 mg/kg or 1.4–3.3 mmol/kg) is then administered orally or intravenously to keep the blood ethanol level within the prescribed range [28,33].

Fomepizole is easier to dose, does not cause drunkenness, and inhibits alcohol dehydrogenase substantially, but it is also more expensive. Ethanol is less expensive, but it is more challenging to dose precisely, necessitates close monitoring of the serum ethanol content, and induces drunkenness that may require acute care monitoring [34,35]. Furthermore, ethanol therapy may result in major problems such as hypoglycemia, changes in awareness level, liver toxicity, or pancreatitis [28,36]. In our cases, it is noted that the patients who survived, patients 4, 5, and 6, were administered with fomepizole in contrast to those who died, while ethanol was given only to patient 3. The fomepizole-treated patients also showed fewer complications and the best outcome when compared with the non-fomepizole-treated patients. In agreement, a case report study had reported that the use of fomepizole prevented vision defects and stabilized vital signs of a 1 year old who ingested alcohol paste with 80% methanol [37]. In contrast, Holzman, Larsen (2021), reported that all cases of methanol poisoning died despite administering fomepizole and hemodialysis [29].

Hemodialysis, including extracorporeal therapy, is another successful treatment for methanol intoxication to correct acidosis and remove methanol from the blood [38]. After being transferred to the ICU department, three of the patients (4, 5, and 6) received hemodialysis while the others did not, due to circulatory collapse. Coma, seizures, early signs of visual impairment, blood pH < 7.15, severe metabolic acidosis, AG > 24 mmol/L, and high serum methanol concentrations are all reasons for hemodialysis [12]. Hemodialysis was not administered to the patients who died (patients 1, 2, and 3) although they met all these criteria, with pH < 7.15, AG > 24 mmol/L, coma, and visual impairment. In contrast, hemodialysis was administered to the patients who survived (cases 4, 5, and 6) although they did not meet all the criteria of hemodialysis. Patients 4, 5, and 6 had vision defects but were conscious. Patient 4 had pH > 7.15, while patients 5 and 6 still had pH < 7.15. Patients 5 and 6 had AG < 24 mmol/L, while patient 4 had AG > 24 mmol/L. However, the patients who survived received hemodialysis sessions while those who died did not and this, side-by-side with the use of fomepizole, enhanced the outcome of those who survived over those who died and showed multi-organ failure. It has been noted that blood pressure increased after the hemodialysis sessions, which is in agreement with a previous case report study [38]. Another retrospective study has reported the beneficial effect of hemodialysis and its rapid effect on the removal of methanol and its toxic metabolites. In this study, 91 patients received hemodialysis and only 3 of them died, in agreement with our study, while all of the surviving patients had hemodialysis sessions and all of those who died did not receive hemodialysis [39].

To treat and correct severe metabolic acidosis, all six patients were given sodium bicarbonates to reverse acidosis, which may also have had a good impact on the conversion of formic acid to formate and the removal of formate, in agreement with other previous studies [26,29,35,38,40]. Inotropic support including noradrenaline, dopamine, and potassium chloride, in addition to mechanical ventilation, were given to the patients who died, trying to overcome circulatory collapse and pulmonary cardiac arrest, but unfortunately, they failed and the patients died from pulmonary cardiac arrest in addition to anorexic brain damage in patient 1 [41,42,43]. Thiamine was used in patients 3, 4, 5, and 6 but we criticize its role as it has very little beneficial effect on methanol poisoning. Its action is beneficial in ethylene glycol poisoning but not methanol, as it promotes the conversion of the toxic metabolite of ethylene glycol, glycolic acid, to α-hydroxy-β ketoadipate [2].

Regarding the outcome of methanol intoxicated patients, it has been noted that three of the patients died while the other three survived. The dead patients have been shown to have had more severe metabolic acidosis with higher AG, electrolytes disturbance, and inappropriate treatments as compared to the surviving patients. The deaths of patients 1–3 were due to pulmonary cardiac arrest, in addition to anorexic brain damage and renal failure in patient 1. All of the cases suffered from visual impairment, but with severe symptoms in the patients who died. On admission, low pH (pH ≤ 7.00), coma, and insufficient ventilation (PCO_2_ ≥ 23 mmHg) were found to be the biggest predictors of a poor prognosis after methanol poisoning; these symptoms were present strongly in the cases of the patients who died [44,45], which is in agreement with a study performed by Gouda, Khattab (2020) [7]. Gouda, Khattab (2020) reported that patients administered ethanol outside of hospital had improved clinical outcomes [7,44]. The previous finding is consistent with the current study where patients who were administered fomepizole survived. Methanol-induced vision loss appears to have a less predictable outcome. However, it has been reported that more than 80% of patients showed an improvement in optic nerve conductivity during the first years of follow-up [45]. In addition, no link has been found between visual sequelae and the type of antidote used, or the mode of hemodialysis used, while only out of hospital ethanol administration appeared to be beneficial, based on follow-up as seen in a recent methanol poisoning outbreak in the Czech Republic [46].

Furthermore, it has been noticed that patients who survived spent more time in the hospital as compared to those who died, which may provide an insight into the correlation between a poor prognosis and inappropriate treatment protocols, where a good prognosis was noticed in patients who followed treatment protocols, including suitable antidotes, in addition to extracorporeal therapy [29,38].

The current study was concerned about the fatal consequences of personnel shortages, testing, and treatment availability (appropriate antidotes and extracorporeal treatments) in KSA, which may become a problem in the event of a large methanol poisoning outbreak based on the findings of this study. A high case fatality rate may stem from a lack of awareness about methanol poisoning treatment among health professionals, as well as late diagnosis of suspicious cases.

## 4. Limitations and Conclusions

One of the major limitations of this study was the inability to confirm methanol poisoning in the blood samples because, despite its importance, it is not a routinely requested test. In all cases, empirical treatment was started based on the patient’s medical history or a high clinical suspicion of methanol poisoning. The lack of awareness about the importance of requesting a concomitant ethanol level measurement as part of the management plan needs to be addressed.

In conclusion, in KSA hospitals with few resources, mass methanol poisoning poses a serious threat. The lack of diagnostic testing and antidotes in the cases presented herein was overcome by an urgent supply of supportive care, antidotes for some cases, and hemodialysis. This study highlights the dangers of items that are sold without any warnings or ingredients information. The dramatic consequences of methanol poisoning outbreaks are not impossible to predict with the available resources. Awareness programs must be provided for health care staff regarding treatment guideline protocols and diagnostic criteria of methanol poisoning to be able to overcome methanol poisoning outbreaks.

## Figures and Tables

**Table 1:** Demographic and admission profiles for the six cases.

	Patient 1	Patient 2	Patient 3	Patient 4	Patient 5	Patient 6
Gender	Male	Male	Male	Male	Male	Male
Age in years	43	26	44	41	32	30
Nationality	Saudi	Indian	Saudi	Nepalese	Indian	Indian
Hospital admission date	16 November 2022	16 November 2022	16 November 2022	4 March 2022	4 March 2022	4 March 2022
Hospital duration	10 h	12 h	10 h	5 d	3 d	3 d
Exposure	Alcohol history	Alcohol history	Colonia ingestion	Alcohol ingestion (colonia)	Alcohol ingestion	Alcohol ingestion (colonia)
Methanol poisoning	Yes	Yes	Yes	Yes	Yes	Yes
Methanol level (mg/dL)	NA	275	NA	NA	NA	NA

NA: data not available.

**Table 2:** Vital signs and clinical laboratory parameters for the six cases.

	Patient 1	Patient 2	Patient 3	Patient 4	Patient 5	Patient 6
Pulse (beats/min)	101	80	138	62	96	82
RR (respirations/min)	20	20	22	20	20	20
BP (mm.Hg)	79/64	98/48	170/116	133/90	143/87	145/95
Temp (°C)	37	37	34.7	36.6	36.6	38.2
SPO_2_ (%)	100	100	99	90	96	95
PCO_2_ (mm.Hg)	45.9	54.2	19	36.4	28	25
Arterial pH	6.56	6.8	7	7.19	7.1	7.1
HCO_3_ (mmol/L)	1.7	1	4	10	11.2	8.5
TLC	18.8	8.47	NA	NA	NA	NA
Hgb (g/dL)	14.8	16	16.4	13	17.1	16
WBC (g/L)	18.8	12.24	11.9	6.34	14.1	17
PLT (g/L)	206	151	342	355	307	389
INR	1.1	NA	1.1	1.14	1.08	NA
AST (U/L)	173	446	48	41	35	19
ALT (U/L)	82	116	81	59	29	12
ALP (U/L)	106	61	109	NA	103	109
Direct bilirubin (mg/dL)	0.16	0.46	0.14	0.2	0.2	0.3
T.protein (g/dL)	5.9	5	NA	6.3	6.6	6.6
Albumin (g/dL)	3.6	2.6	3.7	3.5	3.5	3.6
T.bilirubin (mg/dL)	0.2	0.6	0.3	0.7	0.7	0.7
λGT (U/L)	111	155	NA	137	30	58
RBS (mg/dL)	151	207	261	117 (FBS)	64	81
LDH (U/L)	510	NA	250	NA	NA	NA
Mg (mg/dL)	NA	2.55	1.59	NA	NA	1.7
Ca (mg/dL)	7.8	9.4	9.1	8.4	5.8	8.6
Na (mmol/L)	142	160	143	139	134	142
K (mmol/L)	4.4	1.4	3.2	3.4	3.2	3.2
Cl (mmol/L)	111	122	109	107	103	113
Osmolality serum (Mosmol/kg)	283.5	312	329	274.85	260.7	283.9
Cr (mg/dL)	1.36	1.38	1.11	0.7	0.73	0.77
Urea (mg/dL)	27.82	27.8	26.2	14.98	34	23.54

NA: data not available. RR: respiratory rate, BP: blood pressure, Temp: temperature, SPO_2_: oxygen saturation, PCO_2_: partial pressure of carbon dioxide, HCO_3_: bicarbonate, TLC: total leukocyte count, Hgb: hemoglobin, WBC: white blood cell, PLT: platelet thrombocyte count, INR: international normalized ratio, AST: aspartate aminotransferase, ALT: alanine transaminase, ALP: alkaline phosphatase, λGT: λ glutamyl transferase, RBS: random blood sugar, LDH: lactate dehydrogenase, Mg: magnesium, Ca: calcium, Na: sodium, K: potassium, Cl: chloride, Cr: creatinine.

**Table 3:** Clinical symptoms of methanol poisoning for the six cases.

	Patient 1	Patient 2	Patient 3	Patient 4	Patient 5	Patient 6
Eye:						
- Pupil	Dilated	Dilated	Dilated	Dilated with sluggish response	Dilated	Dilated
- Vision	Visual loss	Non-reactive to light	Lost left eye	Toxic optic nerve damage	Toxic optic nerve damage	Toxic optic nerve damage and blurred vision
Heart	Arrest	Arrest	Tachycardia, arrest	Normal	Normal	Normal
Respiratory	Normal	Mechanical ventilation	Gasping breath, arrest	Normal	Normal	Normal
Temp	Normal	Normal	Hypothermic	Normal	Normal	Fever
Metabolic acidosis	Yes	Yes	Yes	Yes	Yes	Yes
Abdomen	Normal	Soft, no megally	Pain, soft, coffee vomit	Soft lax	Soft lax, epigastric pain, vomiting	Soft lax
Kidney	Acute renal failure	NA	NA	NA	NA	NA
CNS:						
- CT scan	Diffused edema, anorexic damage	Normal	Normal	Normal	Normal	Normal
- Confusion	Yes	Yes	Yes	Yes	Yes	Yes
- Repeated seizures	NA	Yes	Yes	NA	NA	NA
- Coma	Yes	Yes	Yes	No, drowsy	No	No, drowsy

NA: data not available.

**Table 4:** Management courses of the six cases in the emergency department and hospital.

	Patient 1	Patient 2	Patient 3	Patient 4	Patient 5	Patient 6
IV normal saline (D5NS 100 mL/h)	+	+	+	+	+	+
Sodium bicarbonate infusion	+	+	+	+	+	+
Plasma protein fraction infusion	+	-	-	-	-	-
Hemodialysis	-	-	-	+	+	+
Mechanical ventilation	+	+	+	-	-	-
40 mEq KCl IV Intravenous infusion in 500 mL NSS over 4 or 6 h	+	+	+	+	+	+
Fomepizole 15 mg/kg IV as loading sode	-	-	-	+	+	+
Fomepizole 10 mg/kg IV 4 doses/2 days	-	-	-	+	+	+
Thiamine 100 mg IV/stat	-	-	-	+	+	+
Ethanol 600 mg/kg IV infusion	-	-	+-	-	-	-
Omeprazole 40 mg IV daily	+	+	+	+	+	+
Ranitidine 50 mg IV/stat	-	-	+	-	-	-
Diazepam 3 mg IV/stat	-	+	+	-	-	-
sc enoxaparin 4000 iu once a day	+	+	-	-	+	+
Vasopressor noradrenaline	+	+	+	-	-	-
Dopamine HCL 40 mcg/kg every 1 h	+	-	-	-	-	-
Potassium phosphate 21 mmol/day IV	-	+	+	-	-	-
Magnesium sulphate 2 g/day IV	-	+	+	-	-	-
KCl 600 mg tab tid PO	-	-	-	-	+	+

IV: Intra venous, KCl: Potassium chloride.

**Table 5:** Anion and osmolal gaps for the six cases.

	Patient 1	Patient 2	Patient 3	Patient 4	Patient 5	Patient 6
AG (mmol/L)	33.7	38.4	33.2	25.4	23	23.7
Mean ± SD	33.7 ± 2.87 (died)			23.7 ± 1.23 (survived)		
*p*-value	0.037 (sig)					
OG (mmol/kg)	−18.8	−29.4	19.1	−15.0	−23.31	−13
Mean ± SD	19.10 ± 6.04 (died)			15 ± 5.47 (survived)		
*p*-value	0.024 (sig)					

AG: anion gap, OG: osmolal gap. Data are expressed as mean ± SD. *p*-value was calculated to compare between died and surviving cases regarding AG, and OG using independent *t* test, where signifcance was set at *p* < 0.05.

**Table 6:** Outcomes for the six cases.

	Patient 1	Patient 2	Patient 3	Patient 4	Patient 5	Patient 6
Outcome	Dead	Dead	Dead	Improved	Improved	Improved
Causes of death	Post-cardiac arrest	Post-cardiac arrest	Post-cardiac arrest	No	No	No
	Anorexic brain damage	Pulmonary arrest	Pulmonary arrest			

## Data Availability

The data that support the findings of this study are available in the manuscript.
